# Duplex TaqMan Hydrolysis Probe-Based Molecular Assay for Simultaneous Detection and Differentiation of* Burkholderia pseudomallei* and* Leptospira* spp. DNA

**DOI:** 10.1155/2019/9451791

**Published:** 2019-07-02

**Authors:** Mohammad Ridhuan Mohd Ali, Lee Lih Huey, Phiaw Chong Foo, Yuan Xin Goay, Asmaliza S. Ismail, Khairul Mohd Fadzli Mustaffa, Ismail Aziah, Phua Kia Kien, Azian Harun, Nabilah Ismail, Chan Yean Yean

**Affiliations:** ^1^Bacteriology Unit, Infectious Disease Research Centre, Institute for Medical Research, Ministry of Health Malaysia, National Institutes of Health Complex, Bandar Setia Alam, 40170 Shah Alam, Selangor, Malaysia; ^2^Department of Medical Microbiology & Parasitology, School of Medical Sciences, Universiti Sains Malaysia, Health Campus, 16150 Kubang Kerian, Kelantan, Malaysia; ^3^Acarology Unit, Infectious Disease Research Centre, Institute for Medical Research, Ministry of Health Malaysia, National Institutes of Health Complex, Bandar Setia Alam, 40170 Shah Alam, Selangor, Malaysia; ^4^School of Health Sciences, Universiti Sains Malaysia, Health Campus, 16150 Kubang Kerian, Kelantan, Malaysia; ^5^INTI International College Penang, Lebuh Bukit Jambul, Bukit Jambul, 11900 Bayan Lepas, Pulau Pinang, Malaysia; ^6^Institute for Research in Molecular Medicine, Universiti Sains Malaysia, Health Campus, 16150 Kubang Kerian, Kelantan, Malaysia; ^7^Research Policy & Planning Division, National Institutes of Health, Ministry of Health Malaysia, Bandar Setia Alam, 40170 Shah Alam, Selangor, Malaysia; ^8^Hospital Universiti Sains Malaysia, Health Campus, 16150 Kubang Kerian, Kelantan, Malaysia

## Abstract

Melioidosis and leptospirosis, caused by two different bacteria,* Burkholderia pseudomallei* and* Leptospira *spp*.,* are potentially fatal infections that share a very similar spectrum of clinical features and cause significant mortality and morbidity in humans and livestock. Early detection is important for better clinical consequences. To our knowledge, there is no diagnostic tool available to simultaneously detect and differentiate melioidosis and leptospirosis in humans and animals. In this study, we described a duplex TaqMan probe-based qPCR for the detection of* B. pseudomallei* and* Leptospira* spp. DNA. The performance of the assay was evaluated on 20* B. pseudomallei* isolates, 23* Leptospira* strains, and 39 other microorganisms, as well as two sets of serially diluted reference strains. The duplex qPCR assay was able to detect 0.02 pg (~ 4 copies)* Leptospira* spp. DNA and 0.2 pg (~ 25.6 copies)* B. pseudomallei* DNA. No undesired amplification was observed in other microorganisms. In conclusion, the duplex qPCR assay was sensitive and specific for the detection of* B. pseudomallei* &* Leptospira* spp. DNA and is suitable for further analytical and clinical evaluation.

## 1. Introduction

*Burkholderia pseudomallei* and* Leptospira* are two important infectious agents for melioidosis and leptospirosis, respectively [1–3]. The Gram-negative* B. pseudomallei* is recognized as CDC Tier 1 select agent and a Category B Priority Pathogen by the National Institute of Allergy and Infectious Diseases (NIAID), in addition to leptospirosis, which has been added to the Emerging Infectious Diseases category (https://www.niaid.nih.gov/research/emerging-infectious-diseases-pathogens). Both organisms are normally found in the soil and freshwater environment [4, 5]. In addition to their ubiquitous habitats, these organisms routinely infect animals, such as cattle, sheep, and horses. Certain animal classes such as rats may asymptomatically carry* Leptospira. *It is generally accepted that animals are responsible for shedding and maintenance of* Leptospira* and* B. pseudomallei* in the environments, through their urines and faeces [5–7]. Human cases are usually associated with interactions with the contaminated environments [6, 8]. To date, increasing cases of melioidosis and leptospirosis have been reported worldwide, especially in the tropical and subtropical regions [1, 6].

Infections by* B. pseudomallei* and* Leptospira* portray a very similar spectrum of nonspecific clinical presentations including fever, headache, myalgia, and pneumonia [4, 8]. In animals,* B. pseudomallei* infections cause pneumonia with lung abscesses, anorexia, and encephalitis [9]. Meanwhile, animal leptospirosis is characterized by abortion, jaundice, and infertility [10]. Several factors, such as bacterial load, underlying medical conditions, and serotypes increase hosts susceptibility to melioidosis and leptospirosis [1, 11–13]. Furthermore, the risk of dual infection is apparent, as several incidences of melioidosis-leptospirosis coinfections were reported previously [14, 15]. It is possible that many cases may be underdiagnosed when only one between the two tests is considered or available [16].

Early detection of melioidosis and leptospirosis could significantly increase the chances of survival and reduce potential economic loss [17]. Current gold standard for detecting* B. pseudomallei* is by the culture method which requires 2-7 days to grow [18]. Meanwhile, leptospiral antibody titer is detected by the microscopic agglutination test (MAT) that usually requires paired sera and is less useful during acute infection [5]. As both diagnostic methods are time-consuming, a more rapid laboratory assay is urgently needed. To date, several molecular assays have been described for detection of individual* B. pseudomallei* and* Leptospira* from the clinical specimens [5, 18]. However, to our knowledge, none of the reported assays is able to simultaneously detect and distinguish* B. pseudomallei* and* Leptospira *within the same reaction tube. In this study, we developed a duplex qPCR that can detect* B. pseudomallei* and* Leptospira* DNA and evaluated the assay on selected clinical and environmental isolates.

## 2. Materials and Methods

### 2.1. Microorganism Strains and Growth Conditions

A total of 20* B. pseudomallei* strains, 23* Leptospira* strains and 39 other microorganisms isolated from human clinical samples and ATCC strains were used in this study (Table 1). These microorganisms were provided by the Department of Medical Microbiology & Parasitology, School of Medical Sciences, Universiti Sains Malaysia; Makmal Kesihatan Awam Kota Bharu; Universiti Putra Malaysia; and Institute for Medical Research. The bacteria were cultured aerobically in nutrient broth overnight at 37°C on a rotating platform of 180 rpm. Meanwhile,* Leptospira* strains were maintained in EMJH media, incubated at 30°C on rotating platform of 40 rpm, overnight.* Entamoeba histolytica* DNA was obtained directly from School of Health Sciences, Universiti Sains Malaysia.

### 2.2. Isolation of Genomic DNA

DNA was extracted from pure bacterial culture using NucleoSpin® Tissue DNA Extraction kit (MACHEREY-NAGEL GmbH & Co. KG, Germany). The extraction procedure was carried out according to the manufacturer instructions with a minor modification on the final elution step, in which the column was incubated at room temperature for 10 minutes prior to centrifugation at 11 000 ×* g*. Total DNA was quantified using the Eppendorf BioPhotometer (Eppendorf Scientific, Inc., New York, United States) and stored at -20°C until use.

### 2.3. Duplex Real-Time PCR Parameters

The PCR reaction was prepared in a total volume of 20 *μ*L, containing 10 *μ*L 2× SsoAdvanced™ Universal Probes Supermix, 1 *μ*L PCR grade distilled water, 0.2 *μ*M primers, 0.1 *μ*M probes, and 8 *μ*L DNA template. Sequences of oligonucleotides used are listed in Table 2. The oligonucleotides were designed for amplification of the* orf2* region of* B. pseudomallei *type III secretion system (T3SS) and the* rrs* gene of* Leptospira*.

Amplifications were conducted using Biorad CFX96 Touch Real-Time PCR Detection System. Thermal cycling condition included an initial denaturation at 95°C for 5minutes, followed by 50 cycles of 95°C for 30 seconds and 61.3°C for 30 seconds. Baseline threshold for the postamplification analysis was set at 50 (for* B. pseudomallei*) and 25 (for* Leptospira*). Any Cq value ≤40 is considered positive. All the amplification in this study was carried out in triplicate, unless specified otherwise.

### 2.4. Analytical Sensitivity and Specificity

The analytical sensitivity of the assay was carried out using extracted* B. pseudomallei *and* L. interrogans* gDNA, diluted 10-fold ranging from 10 ng/uL to 1 fg/uL. Two microliters of each diluted gDNA were used in the duplex qPCR. Amount of bacterial DNA in each reaction was calculated based on a formula previously described by Aghamollaei* et al*. (2015) [21]. Meanwhile, the assay analytical specificity was determined using 2 *μ*L extracted DNA from other organisms (non-*Leptospira* DNAs and non-*B. pseudomallei*), as listed in Table 1. DNA were extracted using NucleoSpin® Tissue extraction kit.

## 3. Results and Discussions

Despite the availability of several TaqMan hydrolysis probe-based assays for the detection of either* Leptospira* spp. or* B. pseudomallei*, none of the reported assays are able to simultaneously detect both organisms within the same reaction [18, 22]. Availability of such diagnostic tool that is able to detect and differentiate* B. pseudomallei* or* Leptospira* spp. is crucial as both infections portray similar clinical features and yet require different clinical management. In this study, a duplex qPCR for detection of* B. pseudomallei* and* Leptospira* spp. DNA was evaluated. As shown in Table 3, the developed qPCR was able to amplify 0.02 pg (~ 4 copies)* Leptospira* spp. DNA and 0.2 pg (~ 25.6 copies)* B. pseudomallei* DNA, respectively. The sensitivity of the duplex assay for detection of* Leptospira* DNA is comparable to other reported leptospiral probe-based assays that detected between 1 and 20 DNA copies per reaction [23–25]. Meanwhile, for the* B. pseudomallei* detection, the sensitivity was slightly lower than the previously reported assays that amplified 5 and 10 DNA copies per reaction [26–28]. In comparison to the corresponding monoplex assay, the duplex assay had comparable sensitivity for* Leptospira*, but had a reduced sensitivity for* B. pseudomallei* target (0.2 pg in duplex* versus* 0.02 pg in monoplex). Reduced performance of multiplex assay as compared to the monoplex assay has been observed in other molecular studies which are associated with primers competition, primer cross hybridization, and template mispriming [29, 30]. When tested on other microorganisms, no cross amplification was observed (Table 1). The* orf2* region is selected because it is only present in* B. pseudomallei *[31]. Meanwhile, for the leptospiral target, the* rrs* gene is used because the gene is present in multiple copies per* Leptospira* genome [32]. As the current panel included limited coverage of organisms, further validation should include* Burkholderia mallei* and other* Burkholderia cepacia* complex (BCC).

As listed in Table 4, the duplex assay had an efficiency of 101.9% for the detection of* Leptospira* DNA, comparable to the monoplex assay (100.5%). However, for the detection of* B. pseudomallei* DNA, the duplex assay had an efficiency of 86.3%, lower than the monoplex assay (95.9%). The suboptimal efficiency may be attributed to the decreased sensitivity of the assay on* B. pseudomallei* target. In an ideal condition, PCR efficiency should be 90% and above [33]. Further optimization is necessary in order to increase the assay efficiency, especially for the* B. pseudomallei* target. Meanwhile, in terms of linearity, the duplex assays (for* Leptospira* and* B. pseudomallei* DNA detection) had R^2^ values of close to 1. Noticeably, at low copy number, the CV values ranged between 2.8 and 3.8% (Table 3).

Overall, the establishment of a duplex qPCR assay that can detect and differentiate* B. pseudomallei *and* Leptospira *spp. may help the diagnosis of melioidosis and leptospirosis. However, prior to clinical evaluation, further analytical validation, such as intra- and interassay variation, a wider spectrum of microorganisms for specificity testing, higher number of replicates, and optimization of assays are necessary. In addition, incorporation of internal amplification control should be considered because certain types of clinical samples such as whole blood and urine may cause PCR inhibition.

## Figures and Tables

**Table 1:** List of organism used for analytical specificity test.

Organism	Source	No. tested (*n*)	Results in duplex qPCR
*Aspergillus fumigatus*	USM, Malaysia	1	Negative
*Bacillus subtilis*	USM, Malaysia	1	Negative
*Burkholderia cepacia*	USM, Malaysia	6	Negative
*Burkholderia pseudomallei*	USM, Malaysia	20	Positive
*Burkholderia thailandensis*	USM, Malaysia	1	Negative
*Campylobacter jejuni*	USM, Malaysia	1	Negative
*Candida albicans*	USM, Malaysia	1	Negative
*Citrobacter freundii*	USM, Malaysia	1	Negative
*Entamoeba histolytica*	UNAM, Mexico	1	Negative
*Enterococcus faecalis*	USM, Malaysia	1	Negative
*Klebsiella pneumoniae*	USM, Malaysia	1	Negative
*Leptospira biflexa* serovar Patoc	IMR, Malaysia	1	Positive
*Leptospira biflexa* serovar Patoc	UPM, Malaysia	1	Positive
*Leptospira borgpetersenii* Celledoni	IMR, Malaysia	1	Positive
*Leptospira borgpetersenii* serovar Ballum	UPM, Malaysia	1	Positive
*Leptospira fainei* serovar Hurtsbridge	IMR, Malaysia	1	Positive
*Leptospira fainei* serovar Hurtsbridge	UPM, Malaysia	1	Positive
*Leptospira interrogans *serovar Australis	UPM, Malaysia	1	Positive
*Leptospira interrogans *serovar Autumnalis	IMR, Malaysia	1	Positive
*Leptospira interrogans *serovar Bataviae	IMR, Malaysia	1	Positive
*Leptospira interrogans *serovar Bataviae	UPM, Malaysia	1	Positive
*Leptospira interrogans *serovar Canicola	UPM, Malaysia	1	Positive
*Leptospira interrogans *serovar Copenhageni	IMR, Malaysia	1	Positive
*Leptospira interrogans *serovar Hebdomadis	UPM, Malaysia	1	Positive
*Leptospira interrogans *serovar Icterohaemorrhagiae RGA	UPM, Malaysia	1	Positive
*Leptospira interrogans *serovar Javanica	IMR, Malaysia	1	Positive
*Leptospira interrogans *serovar Pomona	IMR, Malaysia	1	Positive
*Leptospira interrogans *serovar Pomona	UPM, Malaysia	1	Positive
*Leptospira interrogans *serovar Pyrogenes	IMR, Malaysia	1	Positive
*Leptospira interrogans *serovar Pyrogenes	UPM, Malaysia	1	Positive
*Leptospira interrogans *serovar Tarassovi	IMR, Malaysia	1	Positive
*Leptospira licerasiae* serovar Varillal	IMR, Malaysia	1	Positive
*Leptospira meyeri* serovar Semaranga	IMR, Malaysia	1	Positive
*Leptospira wolffii*	IMR, Malaysia	1	Positive
*Plasmodium falciparum*	MKA Kota Bharu	5	Negative
*Plasmodium knowlesi*	MKA Kota Bharu	5	Negative
*Plasmodium vivax*	MKA Kota Bharu	5	Negative
*Proteus mirabilis*	USM, Malaysia	1	Negative
*Proteus vulgaris*	USM, Malaysia	1	Negative
*Salmonella* Paratyphi A (ATCC 9150)	ATCC, USA	1	Negative
*Salmonella* Paratyphi B (ATCC BAA 1250)	ATCC, USA	1	Negative
*Salmonella* Paratyphi C (ATCC 9068)	ATCC, USA	1	Negative
*Salmonella* Typhi (ATCC 7251)	ATCC, USA	1	Negative
*Salmonella* Typhimurium (ATCC 14028)	ATCC, USA	1	Negative
*Staphylococcus aureus *	USM, Malaysia	1	Negative
*Staphylococcus saprophyticus*	USM, Malaysia	1	Negative

**Table 2:** List of primers and probes used in this study.

Target	Type	Sequence (5′ →3′)	Source
*Leptospira* spp.	Forward primer	ACTGAGACACGGTCCATACT	[19]
	Reverse primer	TAGTTAGCYGGTGCTTTAGGYA	
	Probe	FAM-ACGGGAGGCAGC-ZEN-AGTTAAGAATCTTGC-IBFQ	

*B. pseudomallei*	Forward primer	CCTGGGAGAGCGAGATGTT	[20]
	Reverse primer	GCTGGATGAGAAGAAAGTCC	
	Probe	TexRed-CCACGCACGGCGGAGATTCT-IBRQ	

**Table 3:** Analytical sensitivity of the duplex qPCR assays.

Copies number	Amount (pg)	Duplex qPCR for *B. pseudomallei*			Copies number	Amount (pg)	Duplex qPCR for *Leptospira *spp.		
		Mean Cq	SD	CV (%)			Mean Cq	SD	CV (%)
2560000	20000	18.41	0.05	0.26	4000000	20000	19.3	0.17	0.86
256000	2000	21.75	0.31	1.43	400000	2000	23.09	0.16	0.67
25600	200	25.34	0.12	0.46	40000	200	27.23	0.04	0.16
2560	20	28.97	0.13	0.44	4000	20	30.67	0.32	1.05
256	2	32.8	0.14	0.44	400	2	34.63	0.08	0.24
25.6	0.2	36.96	1.06	2.88	40	0.2	36.34	0.5	1.37
2.56	0.02	-	-	-	4	0.02	38.59	1.49	3.87
0.256	0.002	-	-	-	0.4	0.002	-	-	-

**Table 4:** PCR efficiency and linearity of the duplex qPCR assays.

Parameter	Target	
	*Leptospira* spp.	*B. pseudomallei*
Slope	-3.2776	-3.7006
Efficiency	101.9%	86.3%
Linearity, R^2^	0.9837	0.9987

## Data Availability

The data used to support the findings of this study are available from the corresponding author upon request.

## Supplementary Materials

Supplementary Materials The standard curves of the duplex qPCR assays for the detection of* B. pseudomallei* DNA and* Leptospira* spp. DNA are illustrated in Figure S1 in Supplementary Material.
