# Supplementary material for: Duplex TaqMan Hydrolysis Probe-Based Molecular Assay for Simultaneous Detection and Differentiation of Burkholderia pseudomallei and Leptospira spp. DNA
Source: Biomed Res Int. 2019 Jul 2;2019:9451791. doi: 10.1155/2019/9451791 (PMC6633960; doi:10.1155/2019/9451791)
Supplement: Supplementary Materials — The standard curves of the duplex qPCR assays for the detection of B. pseudomallei DNA and Leptospira spp. DNA are illustrated in Figure S1 in Supplementary Material. [file 9451791.f1.pdf]

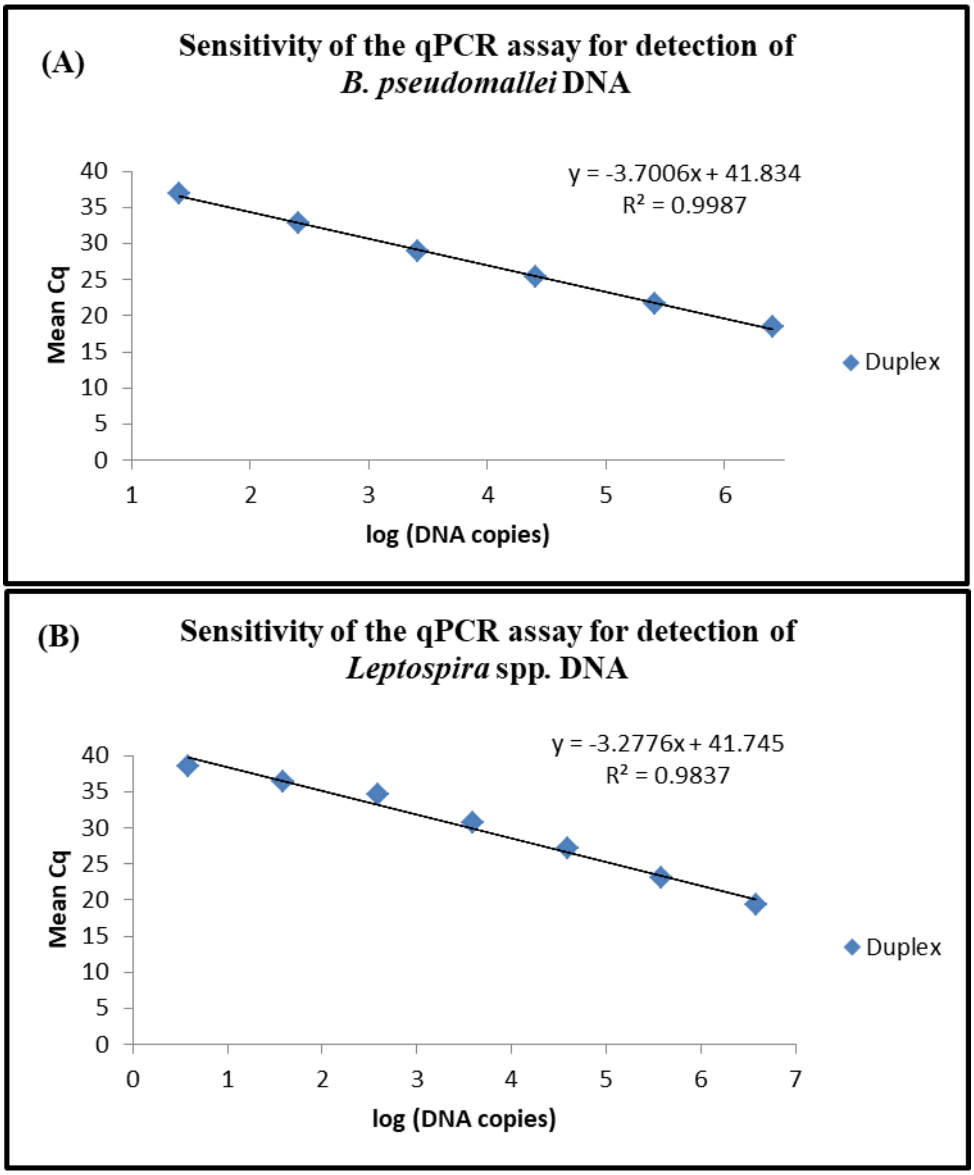

Supplementary Figure 1 Standard curves of the duplex qPCR assays for detection of (A) *B. pseudomallei* DNA and (B) *Leptospira* spp. DNA
